# Chlamydial and gonorrheal neglected sexually transmitted diseases among Pacific Islanders of the Western Pacific Region—A narrative review and call to action

**DOI:** 10.1371/journal.pntd.0011171

**Published:** 2023-03-16

**Authors:** Isabella Catherine Auchus, Mike Kama, Redwan Al-Karim Bhuiyan, Joelle Brown, Deborah Dean

**Affiliations:** 1 Department of Medicine and Pediatrics, University of California San Francisco School of Medicine, San Francisco, California, United States of America; 2 Ministry of Health and Medical Services, Suva, Fiji; 3 Bixby Center for Global Reproductive Health, University of California San Francisco, San Francisco, California, United States of America; 4 Global Health Sciences Institute, University of California San Francisco, San Francisco, California, United States of America; 5 Benioff Center for Microbiome Medicine, University of California San Francisco, San Francisco, California, United States of America; Liverpool School of Tropical Medicine, UNITED KINGDOM

## Abstract

The Pacific Island countries of the Western Pacific Region have some of the highest rates of sexually transmitted *Chlamydia trachomatis* and *Neisseria gonorrhoeae* infections in the world. Despite this, there are few research studies that include Pacific Islanders. We conducted a narrative review of original research and surveys, including World Health Organization and Pacific Community reports, to determine the prevalence, management, and treatment of *C*. *trachomatis* and *N*. *gonorrhoeae* compared to HIV and syphilis from 1980 to 2022. Available epidemiologic data on *C*. *trachomatis* and *N*. *gonorrhoeae* indicated an extremely high prevalence—approximately 30% and 13%, respectively—among Pacific Islanders during this timeframe. These neglected sexually transmitted infections represent a significant burden and health disparity. Robust epidemiologic research is needed to identify modifiable risk factors for designing interventions and control strategies. Appropriate policies along with regional and international advocacy and aid are required to improve reproductive health among these vulnerable, understudied populations to avert preventable infections and sequelae.

## Introduction

**Fig 1:**
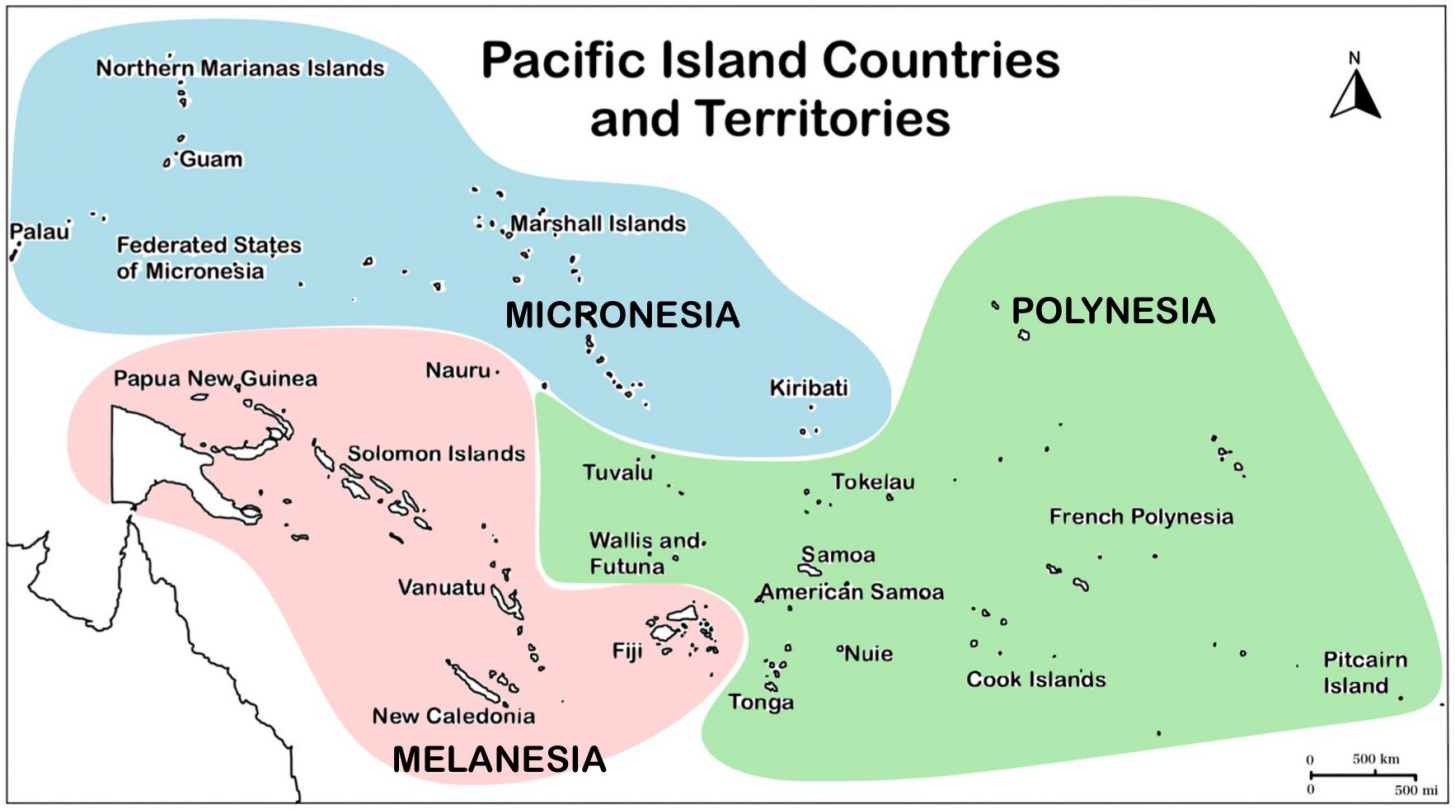
Pacific Island Countries and Territories (PICTs) of the Western Pacific Region. PICTs are grouped by Micronesian, Melanesian, and Polynesian ethnicities. The base layer image from which the figure was initially drawn can be found at https://ojs.wpro.who.int/ojs/index.php/wpsar/article/view/59 (please click on article PDF). WHO uses the Creative Commons Attribution 3.0 IGO license (CC BY 3.0 IGO) with unrestricted use. The link to these terms of use and license information for the base layer image is under the Copyright Notice section at: https://ojs.wpro.who.int/ojs/index.php/wpsar/about.

Some of the highest prevalence and incidence rates of curable sexually transmitted infections (STIs) in the world today are found among Pacific Islanders of the 22 Pacific Island Countries and Territories (PICTs) of the Western Pacific Region (WPR) (Fig 1) [1]. Few studies examining the epidemiology of STIs among these low- and middle-income countries (LMICs) have been performed. PICTs have been consistently excluded from large-scale efforts towards STI surveillance and management, except for human immunodeficiency virus (HIV) and syphilis, with infrequent national or regional surveys [2]. The few studies that have been published have shown a high prevalence of sexually transmitted *Chlamydia trachomatis* and *Neisseria gonorrhoeae* since the late 1980s [3,4]. Two recent studies among nonpregnant and pregnant women have confirmed that the prevalence of both is still high [5,6]. In fact, the World Health Organization (WHO) in 2016 estimated the annual incidence of sexually transmitted *C*. *trachomatis* and *N*. *gonorrhoeae* in the WPR at over 60 M (or 46% of all global cases) and 35 M (or 45% of all global cases), respectively—the highest incidences of chlamydia and gonorrhea for any region in the world [1].

Nearly all LMICs in this region follow WHO guidelines for syndromic management of STIs—a common practice in low-resource countries that relies on signs and symptoms as indicators for treatment [7]. Lack of or inadequate STI testing along with high proportions of asymptomatic infection—up to 80% for chlamydia and 50% for gonorrhea among males and females, respectively—have led to many undiagnosed and untreated cases [8–11]. These untreated STIs can lead to serious health consequences including pelvic inflammatory disease (PID), infertility, ectopic pregnancy, and preterm birth, as well as the burdens of treatment costs, lost wages from illness, psychosocial disruption of families, and increased risk of HIV [12–16].

Inequities and gaps in knowledge are especially harmful to Pacific Islanders who comprise most of the populations living in PICTs. People of Pacific Island descent have historically been grouped into “Asian and Pacific Islander” (API) as a catch-all ethnic category [17,18]. As various Pacific Islander ethnicities are frequently included in this combined category, unique demographic and behavioral risk characteristics may be hidden, preventing a comprehensive understanding of the magnitude and complexity of health issues, such as communicable and noncommunicable diseases, including STIs, which disproportionately affect this population [19–25]. Additionally, Melanesians, Micronesians, and Polynesians within the Pacific Islander ethnic group should be targeted and studied individually to further identify any differences in the epidemiology of STIs among these populations.

We conducted a narrative review of published research studies, nationwide surveys, and national, regional, and international reports and policies that guide STI testing capacity, management, and treatment approaches for *C*. *trachomatis*, *N*. *gonorrhoeae*, HIV, and syphilis in PICTs from 1980 to the present. Available prevalence and incidence data for the four STIs along with fundamental gaps in the literature are presented. We compare the improvements in testing and management that have been achieved for HIV and syphilis in PICTs, but not yet realized for *N*. *gonorrhoeae* and *C*. *trachomatis*, highlighting what could be possible for these STIs with further research, support, and attention. The worldwide, long-standing problem of lack of representation of Pacific Islanders and their ethnicities in research is discussed. Recommendations for research and action are proposed to improve reproductive health for these vulnerable, neglected populations.

## Methods

We conducted a narrative review to summarize what has been published on the prevalence and incidence of *C*. *trachomatis*, *N*. *gonorrhoeae*, HIV, and syphilis among Pacific Islanders in PICTs, identifying knowledge gaps, and tracking and reporting changes in the testing and management of these four STIs. Research articles were included if they were original articles published between January 1980 and April 2022 on the epidemiology of any of these four STIs among Pacific Islanders in PICTs, the population of primary interest due to the known health disparities affecting this population. Articles were excluded if the population studied was not Pacific Islanders living in PICTs and if they were not original epidemiological research studies. Studies were not excluded based on quality or methods, as this would limit a comprehensive understanding of the extent of the prevalence and incidence of these STIs given the paucity of studies and reality that studies conducted in LMICs often do not have access to adequate resources such as those used in higher-income countries or studies in which research costs have been borne by foreign investigators.

Research articles in PubMed were identified using the following search terms: “Oceanic Ancestry Group”[Mesh] OR “Papua New Guinea” OR “Pacific Islander” OR “Micronesia” OR “Melanesia” OR “Polynesia” OR “Fiji” OR “Samoa” OR “Tonga” OR “French Polynesia” OR “Kiribati” OR “New Caledonia” OR “Solomon Islands” OR “Vanuatu” AND “Australasia”[Mesh] OR “New Zealand”[Mesh] OR “Pacific Islands”[Mesh] OR “Pacific States”[Mesh] AND “Sexually Transmitted Diseases/epidemiology”[mesh] OR “Chlamydia Infections/epidemiology”[majr] OR “Chlamydia/ethnology”[majr] OR “Syphilis/epidemiology”[majr] OR “Gonorrhea/epidemiology”[majr] OR “HIV Infections/epidemiology”[majr]) NOT trachoma NOT yaws. Individual Google and PubMed searches were also conducted to find any public data on the subject. Data were extracted and summarized. No statistical analyses were performed.

We also searched Google Scholar and health organization websites such as WHO and Pacific Community for national, regional, and international surveys, reports, and policies on *C*. *trachomatis*, *N*. *gonorrhoeae*, HIV, and syphilis testing and management between 1980 and 2022 for PICTs. Reports identified as relevant by Fijian Ministry of Health and Medical Services officials and the authors were reviewed to provide background information and track changes in the management of these STIs over time.

## Results

We identified 439 published research articles, of which only 55 were identified as original studies published between January 1980 and April 2022 on the epidemiology of *C*. *trachomatis*, *N*. *gonorrhoeae*, HIV, and syphilis. Seven of the 55 studies included Pacific Islanders living in New Zealand and the Pacific United States but were not included in the review because the focus here was on Pacific Islanders residing in PICTs. The remaining 48 studies were conducted solely in PICTs. Seven surveys and reports were also reviewed to provide background information and track changes in the management of the four STIs over time [1,2,26–30].

### Prevalence and incidence data on STIs among Pacific Islanders are severely lacking and are not representative of all PICTs

Of the 48 studies conducted between 1980 and 2022, most included only specific at-risk populations (e.g., pregnant women, female sex workers (FSWs)) with small sample sizes and, therefore, limited generalizability. The majority of these studies were cross-sectional, and none of the studies estimated STI incidence. Published studies were conducted in fewer than half of the PICTs (i.e., Fiji, French Polynesia, Kiribati, New Caledonia, Papua New Guinea (PNG), Samoa, Solomon Islands, Tonga, and Vanuatu) and are therefore not representative of all PICTs in the WPR. Some published studies did not distinguish between the different Pacific Islander ethnicities. Overall, 29 publications included prevalence data on *C*. *trachomatis*, 26 on *N*. *gonorrhoeae*, 25 on HIV, and 27 on syphilis. Study counts do not add up to 48 as some studies tested for multiple STIs. Below, and in Tables 1 and 2, we summarize the prevalence of *C*. *trachomatis*, *N*. *gonorrhoeae*, HIV, and syphilis from the available published literature.

#### C. trachomatis

Between 1980 and 2000, pregnant women attending antenatal clinics had a *C*. *trachomatis* prevalence of over 17% across six PICTs in seven studies; the highest prevalence estimates were reported in Fiji (50%), Samoa (31%), Tahiti (24%), New Caledonia (20%), Vanuatu (22%), and PNG (18%) [4,31–36] (Table 1). From 2000, five studies reported prevalence estimates among pregnant Pacific Islanders. The largest study, conducted in 2008, spanned six PICTs including Fiji, Kiribati, Samoa, Solomon Islands, Tonga, and Vanuatu, and reported a prevalence of 26% among pregnant women under 25 years of age, and 38% among pregnant teens [6]. Three studies conducted in PNG between 2015 and 2016 found a chlamydial prevalence of 11% to 23%, indicating that the burden of *C*. *trachomatis* among pregnant women has not decreased in the country over the past few decades [10,37,38].

**Table 1:** Original research publications on sexually transmitted *C*. *trachomatis* and *N*. *gonorrhoeae* infections in PICTs.

		*Demographics*		*C*. *trachomatis*		*N*. *gonorrhoeae*	
PICTs*	Publication	Age in years	Population	Prevalence	Test†/Specimen Type	Prevalence	Test†/Specimen Type
**Fiji**							
	Svigals 2020 [5]	18–40	Women (Nonpregnant)	18% overall 31% for ages 18–24	Cepheid Xpert CT/NG PCR/vaginal swab	5% overall	Cepheid Xpert CT/NG PCR/vaginal swab
	Gaunavinaka 2014 [44]	14–55	Men with recurrent urethral discharge	2% overall	NAAT/FVU, urethral swab	21% overall	Gram stain, culture/FVU, urethral swab
	Cliffe 2008 [6]	15–44	Women (Pregnant)	29% overall 34% for ages <25	Roche COBAS Amplicor PCR/urine sample	2% overall 3% for ages <25	Roche COBAS Amplicor PCR/urine sample
	Mathai 1998 [34]	-	Women (Pregnant)	20% overall	Chlamydial antigen (Chlamydiazyme ELISA)/endocervical swab	<1% overall	Culture/endocervical swab
	Gyaneshwar 1987 [33]	Mean age of 26.1	Women (Pregnant)	50% for Fijians 38% for Indo-Fijians	Chlamydiazyme immunoassay/vaginal swab	3% for Fijians 1% for Indo-Fijians	Gram stain, culture/vaginal swab
**French Polynesia**							
	Chungue 1988 [4]	M: 17–37 W: 14–45	Men, Women (Pregnant), Bar girls	37% for men 24% for pregnant women 53% for bar girls	Syva MicroTrak DIF, Culture/urethral swabs for men; endocervical swabs for women	59% for men 1% for pregnant women 11% for bar girls	Culture/urethral swabs for men; endocervical swabs for women
**Kiribati**							
	Toatu 2018 [41]	18–33	FSW	25% overall	PCR/urine sample	3% overall	PCR/urine sample
	Cliffe 2008 [6]	15–44	Women (Pregnant)	13% overall 20% for ages <25	Roche COBAS Amplicor PCR/urine sample	0% overall	Roche COBAS Amplicor PCR/urine sample
**New Caledonia**							
	Corsenac 2015 [46]	18–49	Men, Women (Nonpregnant)	9% overall	PCR/urine sample	4% overall	PCR/urine sample
	Morillon 1992 [36]	-	Women (Pregnant)	20% overall	Unknown/serum	-	-
**Papua New Guinea**							
	Hakim 2021 [48]	≥12	MSM, TGW	20% for Port Moresby 19% for Lae 24% for Mt Hagen	PCR, Cepheid Xpert CT/NG/urine sample	10% for Port Moresby 9% for Lae 10% for Mt Hagen	PCR, Cepheid Xpert CT/NG/urine sample
	Vallely 2016 [37]	18–35	Women (Pregnant)	23% overall	PCR/vaginal swab	14% overall	PCR/vaginal swab
	Badman 2016 [10]	Median age 24	Women (Pregnant)	20% overall	Cepheid Xpert CT/NG PCR/vaginal swab	11% overall	Cepheid Xpert CT/NG PCR/vaginal swab
	Wangnapi 2015 [38]	16–49	Women (Pregnant)	11% overall	Bio-Rad CFX96 Real Time PCR/vaginal swab	10% overall	Bio-Rad CFX96 Real Time PCR/vaginal swab
	Vallely 2014 [45]	-	Men, Women (Nonpregnant)	29% overall	Bio-Rad & QuantiFast Probe PCR/cervical swab for women; urethral swab for men with discharge; urine sample for men without discharge	22% overall	Bio-Rad & QuantiFast Probe PCR/cervical swab for women; urethral swab for men with discharge; urine sample for men without discharge
	Bruce 2010 [43]	18–40	FSW	23% overall	PCR/vaginal swab	37% overall	PCR/vaginal swab
	Mgone 2002 [40]	13–50	FSW	31% overall	PCR/vaginal swab	36% overall	PCR/vaginal swab
	Mgone 2002 [51]	Reproductive age	Women (Nonpregnant)	27% overall	PCR/endocervical swab	18% using PCR	PCR/endocervical swab
	Passey 1998 [49]	Reproductive age	Women (Nonpregnant)	-	-	1% using gram stain and culture	Gram stain, culture/vaginal and endocervical swab
	Passey 1998 [9]	15–45	Women (Nonpregnant)	23–27% overall	PCR/endocervical swab	1%-2% overall	Gram stain, culture/vaginal and endocervical swab
	Tiwara 1996 [50]	15–45	Men, Women (Nonpregnant)	25% for men 26% for women	PCR, DIF/FVU sample for men, endocervical swab for women	2% for women Not tested for men	Gram stain, culture/endocervical swab
	Theunissen 1995 [74]	17–43	Women (Pregnant)	15% with PCR 9% with DIF 17% with combined PCR and DIF	PCR, DIF/cervical swabs	-	-
	Hudson 1994 [39]	-	Men, Women (Nonpregnant)	26% for men 27% for women	Syva MikroTrak DIF/urethral swabs for men, endocervical swabs for women	10% of males and 11% of females positive for both Ct and Ng	Gram stain, culture/urethral swabs for men, endocervical swabs for women
	Klufio 1992 [35]	Mean age 25	Women (Pregnant)	18% overall	Syva MikroTrak DIF/endocervical swab	-	-
**Samoa**							
	Walsh 2015 [52]	18–29	Women (Nonpregnant)	36% overall	PCR/urine sample	-	-
	Cliffe 2008 [6]	15–44	Women (Pregnant)	27% overall 41% for ages <25	Roche COBAS Amplicor PCR/urine sample	2% overall 6% for ages <25	Roche COBAS Amplicor PCR/urine sample
	Sullivan 2004 [31]	15–48	Women (Pregnant)	31% overall	PCR/intravaginal tampon sample	3% overall	PCR/intravaginal tampon sample
	Ushijima 1990 [57]	10–20, adults	Men, Women, unknown P/NP	39% for IgG 13% for IgA	Denka Seiken ELISA/serum	-	-
**Solomon Islands**							
	Marks 2015 [53]	16–49	Women (Nonpregnant)	20% overall 28% for ages <30	BD-Probetec SDA/vaginal swab	5% overall	BD-Probetec SDA/vaginal swab
	Cliffe 2008 [6]	15–44	Women (Pregnant)	6% overall 7% for ages <25	Roche COBAS Amplicor PCR/urine sample	<1% overall	Roche COBAS Amplicor PCR/urine sample
	Stokes 1982 [3]	-	Women (Pregnant)	-	-	9% overall	Microscopy/endocervical swab
**Tonga**							
	Cliffe 2008 [6]	15–44	Women (Pregnant)	15% overall 28% for ages <2	Roche COBAS Amplicor PCR/urine sample	3% overall 5% for ages <25	Roche COBAS Amplicor PCR/urine sample
**Vanuatu**							
	Veronese 2015 [47]	Median age 25–26	MSM, TGW	18% overall	BD ProbeTec NAAT/rectal swabs	9% overall	BD ProbeTec NAAT/rectal swabs
	Van Gemert 2014 [42]	≥18	FSW	37% overall	BD ProbeTec NAAT/vaginal swabs	17% overall	BD ProbeTec NAAT/vaginal swabs
	Cliffe 2008 [6]	15–44	Women (Pregnant)	13% overall 20% for ages <25	Roche COBAS Amplicor PCR/urine sample	2% overall 4% for ages <25	Roche COBAS Amplicor PCR/urine sample
	Sullivan 2003 [32]	15–46	Women (Pregnant)	22% overall	PCR/intravaginal tampon sample	6% overall	Roche COBAS Amplicor/intravaginal tampon sample

*No original research study data were available from the following PICTs: Cook Islands, Federated States of Micronesia, Guam, Marshall Islands, Nauru, Niue Island, Palau, Pitcairn, Tokelau, Tuvalu, Wallis, and Futuna.

†Several studies did not report specimen type, what was measured or measurements used, companies that produced the test or test names. All test details that were included in publications are included in the table.

DIF, direct immunofluorescence; FSW, female sex worker; MSM, men who have sex with men; TGW, transgender women; FVU, first void urine; NAAT, nucleic acid amplification test; PCR, polymerase chain reaction; SDA, strand displacement amplification assay.

*C*. *trachomatis* prevalence estimates have been the highest for FSW and other “high-risk” populations such as STI clinic attendees, “bar girls,” men who have sex with men (MSM), and transgender women (TGW). In the 1980s, estimates of prevalence among FSW ranged from 27% in PNG to 53% in Tahiti [4,39,40]. Since 2010, the prevalence among FSW ranged from 25% to nearly 37% [41–43]. Studies in 2014 and 2015 found a chlamydial prevalence of 29% among men attending STI clinics in PNG and Fiji [44–46]. Among TGW and MSM, the prevalence of *C*. *trachomatis* was 18% in Vanuatu in 2015 and 24% in PNG in 2021, respectively [47,48].

Five studies of nonpregnant women from the general population during the 1990s found a chlamydial prevalence of 17% to 26% with the highest prevalence (≥26%) being among women in rural areas [9,39,49–51]. Several studies from PNG reported high coinfection rates: Over 10% of women had more than one STI including *C*. *trachomatis*, *N*. *gonorrhoeae*, syphilis, and *Trichomonas vaginalis* [9,39,49,51]. In 2015, chlamydial prevalence was 36% in women aged 16 to 30 years in the Solomon Islands, while young Samoan women had a prevalence of 42% [52,53]. In 2020, 18% of nonpregnant Fijian women aged 18 to 40 years attending Ministry of Health and Medical Services clinics tested positive for *C*. *trachomatis*, while nearly 31% under 25 years were infected [5].

Only six published studies, of which three were from PNG and one each from French Polynesia, New Caledonia, and Samoa, included men from the general population. One reported a prevalence of 25% among men in the 1990s in PNG [50]. The most recent study among men from the general population was published in 2012 and found a chlamydial prevalence of 8% in New Caledonia [46].

#### N. gonorrhoeae

Before 2000, gonorrheal prevalence among pregnant women in PICTs ranged from <1% to 9% (Table 1) [3,4,31–34]. Since 2000, the prevalence of gonorrhea has continued to trend lower than chlamydia but has not decreased; the prevalence across seven PICTs, including Fiji, Kiribati, Samoa, Solomon Islands, Tonga, Vanuatu, and PNG, ranged from 2% to 14%, with the highest prevalence in PNG [6,10,37,38].

Studies conducted before 2000 in PICTs among “high-risk” populations found a gonorrheal prevalence of 11% among bar girls and 36% among FSW in French Polynesia and PNG, respectively [4,40]. Among men attending STI clinics in French Polynesia, 59% were infected [4]. Since 2000, two of three studies found that FSW had a prevalence of over 17% with estimates as high as 37% in PNG in 2010 [41–43]. A 2014 study that included both men and women in PNG found a gonorrheal prevalence of 22% among STI clinic attendees [45]. In 2014, 33% of men attending clinics in Fiji for recurrent urethral discharge were diagnosed with gonorrhea [44]. Two studies from PNG and Vanuatu found a 10% gonorrhea prevalence among MSM and TGW in 2015 and 2021, respectively [47,48].

Since 1980, only four studies have measured the prevalence of gonorrhea among men and women from the general population. In 2002, rural nonpregnant women in PNG had a gonorrheal prevalence of 18% [51]. In 2015, three studies reported a gonorrhea prevalence of 5% among women in the Solomon Islands and Fiji and 4% among men in New Caledonia [5,46,53].

#### HIV

The majority of studies up to the early 2000s found an HIV prevalence of zero to <1% among Pacific Islanders, including men and pregnant and nonpregnant women (Table 2) [6,31,32,34,54–61]. The highest HIV prevalence was found among men and women attending an emergency department in PNG (18%) followed by FSW (10%) who had more than a 10-fold higher HIV prevalence than the general population [40,62]. For young FSW in PNG, HIV prevalence was 16% [43]. Between 2010 and 2020, FSW in Vanuatu and Kiribati were found to have an HIV prevalence of zero, while FSW in PNG had a prevalence of 15% [41,42,63–65]. Studies enrolling MSM and TGW during this time found an HIV prevalence ranging from zero in Vanuatu to 8% in Port Moresby, the capital of PNG [47,66]. Pregnant women in PNG and the Solomon Islands had an HIV prevalence of <1% [37,53]. In Fiji, there were only 725 confirmed HIV cases from 1989 to 2016 [67]. Since 2015, no studies on HIV prevalence have been published in 18 of the 22 PICTs.

**Table 2:** Original research publications on HIV and syphilis in PICTs.

		*Demographics*		*HIV*		*Syphilis*	
PICTs*	Publication	Age in years	Population subgroup	Prevalence	Test†/Specimen Type	Prevalence	Test†/Specimen Type
**Fiji**							
	Gaunavinaka 2014 [44]	14–55	Men with recurrent urethral discharge	-	-	5% overall	VDRL, TPHA/serum
	Cliffe 2008 [6]	15–44	Women (Pregnant)	0% overall	Abbott Determine HIV test kits, Fujirebio Serodia HIV test kits; Positives confirmed by WB/serum	3% overall	TPPA and FTA tests or VDRL and TPPA; IgG conjugate from Dako Corp used to confirm reactive TPPA samples/serum
	Washington 2008 [56]	-	Women (Pregnant)	<1% overall	Fujirebio Serodia HIV-1/2 Antibody detection test; Positives confirmed with WB/serum	-	-
	Mathai 1998 [34]	-	Women (Pregnant)	0% overall	Fujirebio Serodia HIV particle agglutination test/serum	8% overall	VDRL, TPPA/serum
	Gyaneshwar 1987 [33]	Mean age of 26.1	Women (Pregnant)	-	-	14% for Fijians 2% for Indo-Fijians	VDRL, TPHA/serum
**Kiribati**							
	Toatu 2018 [41]	18–33	FSW	0% overall	Abbott Determine HIV test kits/serum	6% overall	Abbott Determine test kits; TPPA, FTA used for confirmatory/serum
	Cliffe 2008 [6]	15–44	Women (Pregnant)	0% overall	Abbott Determine HIV test kits, Fujirebio Serodia HIV test kits; Positives confirmed by WB/serum	2% overall	TPPA and FTA tests or VDRL And TPPA; IgG conjugate from Dako Corp used as confirmatory for reactive TPPA samples/serum
**New Caledonia**							
	Corsenac 2015 [46]	18–49	Men, Women (Nonpregnant)	-	-	<1% overall	TPHA, RPR/serum
	Guerrier 2013 [55]	Mean age 25	Women (Pregnant)	0% overall	ELISA; Positives confirmed by WB/serum	2% overall	RPR (BioRad), reactive samples tested with TPHA (Siemens)/serum
	Ménard 2001 [59]	-	Women (Pregnant)	0% overall	Unknown/serum	7%-12%	Unknown/serum
**Papua New Guinea (PNG)**							
	Hakim 2021 [65]	≥12	FSW	15% overall	Abbott Determine HIV-1/2, Stat-Pak HIV-1/2, GeneXpert HIV viral load assay/serum	-	-
	Hakim 2021 [48]	≥12	MSM, TGW	-	-	6% overall	Chembio DPP Syphilis Assay/serum
	Hakim 2020 [63]	≥12	FSW	15% overall	Abbott Determine HIV-1/2, Stat-Pak HIV-1/2, GeneXpert HIV viral load assay/serum	-	-
	Hakim 2019 [66]	≥12	MSM, TGW	9% for Port Moresby 7% for Lae 1% for Mt Hagen	Abbott Determine HIV-1/2, Stat-Pak HIV-1/2, GeneXpert HIV viral load assay/serum	4% for Port Moresby 9% for Lae 3% for Mt Hagen	Chembio DPP Syphilis Assay/serum
	Vallely 2016 [37]	18–35	Women (Pregnant)	1% overall	Abbott Determine HIV-1/2, Stat-Pak HIV-1/2/serum	2% overall	Abbott Alere Bioline anti-TP 3.0, Confirmatory RPR/serum
	Badman 2016 [10]	Median age 24	Women (Pregnant)	2% overall	Abbott Determine HIV-1/2, Stat-Pak HIV-1/2/serum	4% overall	Abbott Alere Bioline anti-TP 3.0, Confirmatory RPR/serum
	Wand 2015 [64]	-	FSW	7% overall	Abbott Determine HIV1/2 strip, Fujirebio Serodia HIV assay, Ogenics Enzyme Immunoassay Immunocomb HIV1/2 (Ogenics); Discrepant samples tested with p24 antigen ELISA (BioRad)/serum	6% overall	RPR, TPHA/serum
	Wangnapi 2015 [38]	16–49	Women (Pregnant)	-	-	1% overall	Syphicheck WB rapid test, RPR, TPPA/serum
	Vallely 2014 [45]	-	Men, Women (Nonpregnant)	3% overall	Fujirebio Serodia HIV test kits, Abbott Alere/serum Immunocomb enzyme immunoassay	12% overall	RPR/serum
	Bruce 2010 [43]	18–40	FSW	16% overall	Fujirebio Serodia HIV test kits, Capillus HIV test kits/serum	33% overall	VDRL, TPHA/serum
	Suligoi 2005 [54]	-	Rural Melanesian adults	<1% overall	ELISA Vironostika HIV-1 Uniform II plus O/serum	-	-
	Curry 2005 [62]	10–69	Men, Women, seen in the emergency department	18% overall	Abbott Determine HIV test kits, Fujirebio Serodia HIV test kits, Capillus HIV test kits/serum	-	-
	Mgone 2002 [40]	13–50	FSW	10% overall	Vironostika HIV ELISA, ImmunoComb II HIV 1 and 2 BiSpot, Fujirebio Serodia HIV test kits, Capillus HIV test kits/serum	32% overall	VDRL, TPPA/serum
	Passey 1998 [49]	Reproductive age	Women (Nonpregnant)	-	-	4% overall	RPR, TPHA/serum
	Tiwara 1996 [50]	15–45	Women (Nonpregnant)	-	-	5% overall	RPR, TPHA/serum
	Hudson 1994 [39]	-	Men, Women (Nonpregnant)	-	-	5% for men 12% for women	VDRL, TPHA, FTA-abs, TIP/serum
	Yamaguchi 1993 [60]	-	Villagers	0% overall	Particle agglutination test/serum	-	-
	Tawai 1982 [70]	-	Patients at STD clinic	-	-	9% overall	VDRL, TPPA/serum
**Samoa**							
	Cliffe 2008 [6]	15–44	Women (Pregnant)	0% overall	Abbott Determine HIV test kits, Fujirebio Serodia HIV test kits; Positives confirmed with WB/serum	0% overall	TPPA and FTA tests or VDRL And TPPA; IgG conjugate from Dako Corp used as confirmatory for reactive TPPA samples/serum
	Sullivan 2004 [31]	15–48	Women (Pregnant)	0% overall	Abbott ELISA/serum	<1% overall	RPR/serum
	Ushijima 1990 [57]	10–20, adults	Men, Women	0% overall	Abbott ELISA, gelatin PA, WB/serum	-	-
**Solomon Islands**							
	Marks 2015 [53]	16–49	Women (Nonpregnant)	0% overall	Abbott Alere Determine fourth-generation antibody-antigen assay/serum	4% overall	RPR, TPHA/serum
	Cliffe 2008 [6]	15–44	Women (Pregnant)	0% overall	Abbott Determine HIV test kits, Fujirebio Fujirebio Serodia HIV test kits; Positives confirmed with WB/serum	10% overall	TPPA and FTA tests or VDRL And TPPA; IgG conjugate from Dako Corp used as confirmatory for reactive TPPA samples/serum
**Tonga**							
	Cliffe 2008 [6]	15–44	Women (Pregnant)	0% overall	Abbott Determine HIV test kits, Fujirebio Serodia HIV test kits; Positives confirmed with WB/serum	3% overall	TPPA and FTA tests or VDRL And TPPA; IgG conjugate from Dako Corp used as confirmatory for reactive TPPA samples/serum
	Ushijima 1990 [57]	10–20, adults	Men, Women	0% overall	Abbott ELISA, gelatin PA, WB/serum	-	-
**Vanuatu**							
	Veronese 2015 [47]	Median age 25–26	MSM, TGW	0% overall	Abbott Determine Rapid HIV test; Reactive samples confirmed with rapid tests Insti & Unigold/serum	3% overall	RPR, Abbott Alere Determine Syphilis TP/serum
	Van Gemert 2014 [42]	≥18	FSW	0% overall	Abbott Determine Rapid test kits; Reactive samples confirmed with rapid tests Insti & Unigold/serum	4% overall	RPR, Abbott Alere Determine Syphilis TP/serum
	Cliffe 2008 [6]	15–44	Women (Pregnant)	0% overall	Abbott Determine HIV test kits, Fujirebio Serodia HIV test kits; Positives confirmed with WB/serum	3% overall	TPPA and FTA tests or VDRL And TPPA; IgG conjugate from Dako Corp used as confirmatory for reactive TPPA samples/serum
	Sullivan 2003 [32]	15–46	Women (Pregnant)	0% overall	ELISA/serum	2% overall	RPR, TPHA/serum
**Various Island Nations**							
	Brindle‡ 1988 [61]	-	Men, Women	0% overall	ELISA (Wellcome)/serum	-	-

*No original research study data were available from the following PICTs: Cook Islands, Niue Island, Pitcairn, Tokelau, Wallis and Futuna. The “Various Island Nations” in the last row refer to Guam, PNG, Vanuatu, French Polynesia, Palau, Federated States of Micronesia, Kiribati, Nauru, Marshall Islands, and Tuvalu.

†All sample types, measurements, test names, and vendors that were noted in the publications are included.

‡This study tested for HIV in the following PICTs: Guam, PNG, Vanuatu, French Polynesia, Palau, Federated States of Micronesia, Kiribati, Nauru, Marshall Islands, and Tuvalu. The prevalence of HIV was found to be 0% in all locations.

ED, emergency department; FSW, female sex worker; MSM, men who have sex with men; TGW, transgender women; FTA, fluorescent treponemal antibody absorption test; PA, particle agglutination; RPR, rapid plasma regain; TPHA, *Treponema pallidum* hemagglutination test; TPPA, *Treponema pallidum* particle agglutination; VDRL, venereal disease research laboratory; WB, western blot.

#### Syphilis

The most recent syphilis prevalence estimates from the general population, including men and both pregnant and nonpregnant women, are from 2014/2015 and ranged from <1% in New Caledonia to 12% in PNG (Table 2) [10,37,45,46,53]. Recent studies among other “higher-risk” populations found a syphilis prevalence of 6% in 2020 among MSM and TGW in PNG and 6% among FSW in Kiribati in 2018 [41,65]. One study from New Caledonia in 2013 reported that active syphilis was highest among people of Melanesian ethnicity [55].

### Historical and current approaches to STI testing in PICTs

Below, we describe the historical and current approaches to STI testing and surveillance in PICTs, beginning with HIV and syphilis and then contrasting that to current approaches to testing and surveillance for chlamydia and gonorrhea.

#### HIV

In the early 1980s, the spread of HIV, which causes acquired immunodeficiency syndrome (AIDS), was a great concern throughout the region. This was particularly the case in PNG, which had many of the earliest cases with the highest incidence and prevalence in the WPR [2,68]. Much of the epidemiologic data on HIV in PICTs have historically come from surveillance programs at antenatal care clinics and screening of donated blood products [26,68]. Since the early 2000s, rapid HIV testing programs and research studies have expanded to wider at-risk populations, including FSW.

During the 1980s and 1990s, HIV prevalence studies typically used rapid particle agglutination HIV test kits or western blot in local clinics (Table 2). In the 2000s, there was an increase in the use of ELISA tests due to their improved accuracy and adoption in higher-resource countries [31,32,54,55,69]. Since the early 2010s, more studies reported using a rapid antibody/antigen combination as the initial test followed by additional rapid antibody tests if the first result was positive [6,37,41,42,47,48,63,64]. Foreign research efforts significantly expanded the capabilities for HIV testing in PICTs given the cost and limited laboratory and personnel resources in most PICTs [6,55–57].

The current standards for HIV testing vary across and within PICTs. Larger urban hospitals have either established or are beginning to implement HIV testing algorithms. Many PICTs use the recommended algorithm validated for the Pacific, which includes a rapid antibody/antigen screening test followed by two additional rapid antibody tests to confirm reactive samples [26,27]. Although Fiji has a higher gross national income than most other PICTs, HIV testing algorithms differ by subdivisional and divisional hospitals due to differences in resources [27,56].

#### Syphilis

Similar to HIV, interest in syphilis testing began around the 1980s due to concerns about coinfection with HIV, congenital syphilis, high mortality associated with chronic infection, and severe irreversible neurological and cardiac diseases [33,70]. Even with the increase in syphilis testing over the past few decades, testing capabilities in PICTs are not uniform and often inadequate [33,70]. Several PICTs use nontreponemal tests, such as a rapid plasma reagin (RPR) or venereal disease reference laboratory (VDRL) tests, for initial screening, and additional confirmatory treponemal tests like the *T*. *pallidum* particle agglutination (TPPA) test, when available [31,32,34,40]. Since 2017, syphilis testing has undergone significant improvement due to an increase in point-of-care (POC) tests, such as Standard Diagnostics Bioline HIV/Syphilis test, an immunochromatographic assay for the detection of antibodies, and nationwide efforts across PICTs [71].

#### Chlamydia trachomatis and Neisseria gonorrhoeae

The efforts in PICTs to improve testing for and surveillance of HIV and syphilis have unfortunately not been replicated for *C*. *trachomatis* and *N*. *gonorrhoeae*. This is due to several reasons, including a lack of accurate, affordable tests [72]. Neither *C*. *trachomatis* nor *N*. *gonorrhoeae* are routinely tested for or reported in most PICTs. Instead, the majority of PICTs rely on WHO criteria for syndromic management of these STIs [7]. Where testing is available, it usually includes microscopy, cultures, and antigen/antibody tests that lack sensitivity. Other barriers to testing include the lack of healthcare personnel to collect the samples and patients’ unwillingness to be tested due to perceived stigma and/or lack of acceptability of anogenital and anorectal screening, particularly among FSW and MSM [31,32,42,48]. Therefore, what is currently known about the epidemiology of sexually transmitted *C*. *trachomatis* and *N*. *gonorrhoeae* in PICTs comes from select populations, such as young pregnant women or FSW, and small research studies where the costs of testing were borne by foreign investigators [3,4,6,10,31–38,40–43,47,48].

Prevalence studies beginning in the 1980s used gram stains and cultures for *N*. *gonorrhoeae* and ELISAs or other immunofluorescence tests directed against *C*. *trachomatis* antigens that lacked sensitivity [3,4,33,57,73]. Throughout the 1990s, these lower-cost tests continued to be used [34,35,39,74]. Studies from the mid-1990s in PNG were the first in the region to report the detection of *C*. *trachomatis* in urine using polymerase chain reaction (PCR) tests, which are more sensitive than immunofluorescence assays for detecting infection [9,49,50]. For *N*. *gonorrhoeae*, often a combination of microscopy and culture were used for diagnosis as well as for determining infection prevalence [9,34,50]. Starting in the 2000s, nucleic acid amplification tests (NAATs) became the gold standard for detecting *C*. *trachomatis* and *N*. *gonorrhoeae* in urine and urogenital swabs because of their high sensitivity (90% to 100%) and specificity (98% to 100%) [6,31,32,43,75,76]. However, their use has been limited in PICTs because of the high costs of tests, the need for expensive equipment and trained personnel, limited certified laboratories and/or space for performing tests, and supply chain issues related to the availability of both collection and detection kits.

### STI management in PICTs varies by country and priorities

The WHO Regional Office for the Western Pacific periodically releases treatment guidelines for STIs in the WPR [29]. Below, we describe the current approaches to treatment, beginning with HIV and syphilis and then contrasting that to the current standard of care for chlamydia and gonorrhea.

#### HIV

National HIV management plans in PICTs are currently being developed and are at various stages of implementation [2]. The current standard of care for HIV treatment worldwide includes antiretroviral therapy (ART), which is used in PICTs where available free of cost [30]. The first-line ART recommendation for adults, adolescents, and children is triple therapy consisting of one NNRTI and two NRTIs, typically tenofovir, lamivudine, and efavirenz [27]. However, suboptimal use of health services due to the lack of accessible clinics and hospitals has resulted in missed follow-up visits by Pacific Islanders. This can lead to poor adherence to ART, faster disease progression, and increase in the spread of HIV [31,57,58].

#### Syphilis

Management of syphilis in PICTs is also guided by WHO recommendations and the availability of resources [28]. First-line treatment for syphilis infection is intramuscular (IM) benzathine penicillin G (BPG), with alternatives being procaine penicillin, doxycycline, ceftriaxone, azithromycin, and erythromycin. From early 2000 to 2019, four research studies from Vanuatu, Samoa, PNG, and the Solomon Islands investigated syphilis in pregnant women and neonates. While most followed WHO recommendations, in PNG, only 84% of neonates were treated; 8% died and 8% were not treated because results were not provided before labor or available prior to discharge [31,32,77,78].

#### Chlamydia trachomatis and Neisseria gonorrhoeae

Progress in the management of *C*. *trachomatis* and *N*. *gonorrhoeae* in PICTs has not improved as much as for HIV and syphilis. Given the limited availability of tests for chlamydia and gonorrhea, the reliance on syndromic management, and the lack of nationwide surveillance efforts, the actual prevalence and incidence of infection and disease sequelae remains largely unknown and has, therefore, not risen to the appropriate level of concern [41].

PICTs follow WHO guidelines for syndromic management of presumptive chlamydia and gonorrhea, which allows same-day treatment of STIs based on patient signs and symptoms. These guidelines are not uniformly adopted but based on country-specific differences such as personnel and antibiotic availability and antibiotic resistance patterns for gonorrhea, if known [7,28]. A high proportion of sexually transmitted *C*. *trachomatis* and *N*. *gonorrhoeae* infections in men and women are asymptomatic and, therefore, go undiagnosed and untreated with the potential for continued transmission [5,8–11]. Various studies from PICTs have shown that up to 80% of women with chlamydia were asymptomatic, while 100% of pregnant women with gonorrhea were asymptomatic [3,5,10]. In a 2020 study of 103 nonpregnant women in Fiji infected with *C*. *trachomatis*, only 11 met the criteria for syndromic management [5]. Efforts to develop new algorithms to improve syndromic management have been made but lack evidence for use [9,49].

Treatment regimens based on syndromic management for chlamydia and gonorrhea include antibiotics for both the patient and partner(s) without the opportunity for a test of cure [3,10]. Antibiotics used to treat chlamydia include erythromycin, azithromycin, or doxycycline, the latter of which requires twice daily dosing for seven days, which carries the risk of noncompliance [31,32,35,44]. For gonorrhea, a dose of IM ceftriaxone plus a dose of oral azithromycin is recommended [7]. However, as mentioned above, not all antibiotics are available.

Data from the 2014 WHO Global Gonococcal Antimicrobial Surveillance Programme show decreased susceptibility or resistance to ceftriaxone in five of seven surveyed WPR countries, high levels of ciprofloxacin resistance in nearly all countries within the WPR, isolates with resistance to azithromycin in seven of nine surveyed regional countries (Japan, Hong Kong, Mongolia, Australia, New Zealand, Singapore, and Vietnam), and highly prevalent penicillin resistance in the majority of the WPR [79]. However, most PICTs do not consistently perform susceptibility testing or report these data, which is concerning because PICTs could become a source for multidrug-resistant *N*. *gonorrhoeae* with the risk of global spread [79,80].

## Conclusions

Published data on the prevalence and incidence of STIs among Pacific Islanders are severely lacking in PICTs. Only 31 studies have been published over the past four decades on sexually transmitted *C*. *trachomatis* and/or *N*. *gonorrhoeae* infections, resulting in large gaps in our understanding of the burden of these STIs among PICTs for both general and high-risk populations. The majority of these publications have had small sample sizes, studied specialized populations (e.g., pregnant women, FSW), and were conducted in only a few PICTs, resulting in data that are not representative of PICTs as a whole. These limited data suggest that the prevalence of these infections is not decreasing, although the prevalence varies among different subgroups of the populations and by country. Additionally, inconsistent reporting of the types of samples, measurements, or specific test types in these studies is another limitation of this body of literature, which makes it difficult to accurately quantify STI prevalence among these populations. This is especially troublesome as there are substantial economic and health burdens associated with STIs and their sequelae, including the costs of treatment, lost wages from illness, detrimental psychosocial impacts on families, and sequelae such as PID, preterm birth, and infertility [81,82]. Indeed, PICTs already have some of the highest rates of infertility in the world with over 3% for primary infertility and over 11% for secondary infertility [83]. Untreated STIs are likely a primary contributor [5,84]. Furthermore, there are no incidence data, making it difficult to identify modifiable risk factors to guide appropriate interventions and prevention strategies. Research on sexually transmitted *C*. *trachomatis* and *N*. *gonorrhoeae* in LMICs in the WPR should be a high priority for research, not only because of the severe sequelae of these infections, but also because they are known risk factors for HIV infection [1]. This is a perilous situation for a region that is vulnerable to STIs due to an economy primarily built on tourism.

While we made every effort to include all available published STI data from PICTs, our results could potentially overestimate the STI prevalence if publication bias prevented the publication of studies that found a low STI prevalence in PICTs. We also found very few published STI studies that explicitly included Pacific Islander ethnicities, making it difficult to fully comprehend the extent of health disparities related to STIs among these populations. We recommend that future health research include and report Pacific Islander ethnicities to ensure equitable allocation of healthcare resources and interventions within PICTs. Where possible, the collection of original data should allow for the analysis of Pacific Islanders apart from Asians, but also for combining of these groups when the numbers of Pacific Islanders are too small to reveal any significant associations.

The exceptionally high prevalence of sexually transmitted *C*. *trachomatis* and *N*. *gonorrhoeae* among Pacific Islanders in PICTs must be recognized as an urgent public health problem and prioritized at local, national, and international levels. The success that LMICs in the WPR have had with HIV and syphilis testing, laboratory capacity, and surveillance are encouraging examples of what is possible for tackling *C*. *trachomatis* and *N*. *gonorrhoeae*. Based on our knowledge of policies in PICTs and a review of the STI literature, we summarize and suggest several priorities for research and action in the Key Learning Points. International attention, advocacy, and aid are also critical to leverage resources and bolster efforts to prioritize the reproductive health of these vulnerable Pacific Islander populations living in the WPR.

**Key Learning Points**

- The extremely high prevalence of *C*. *trachomatis* and *N*. *gonorrhoeae* among Pacific Islanders in PICTs provides the foundational knowledge to promote changes in national and regional policies to expand STI screening and public health reporting not just for the general population, but also for prenatal, MSM, TGW, FSW, adolescents, and young adult populations.
- Capacity building for STI testing, *N*. *gonorrhoeae* susceptibility testing, and the development of inexpensive, rapid, sensitive, and specific POC tests for *C*. *trachomatis* and *N*. *gonorrhoeae* are a priority in order to treat those who are actually infected, monitor the burden of STIs in the region, and inform efforts to allocate resources and develop prevention campaigns.
- Pacific Islanders and their distinct island ethnicities are essentially erased by their aggregation with other ethnicities in research studies and surveys. Distinct Pacific Islander ethnicities should be included and reported in research studies to identify differences in the epidemiology of sexually transmitted *C*. *trachomatis* and *N*. *gonorrhoeae* infections to focus healthcare initiatives and equitable resource distribution.
- Regional and international support and advocacy for the sexual and reproductive health of Pacific Islanders and inhabitants of PICTs are critical to decrease STIs and thereby improve disease prevention that will also serve as a control for sequelae as well as regional and global transmission.

**Top Five Papers**

- Cliffe SJ, Tabrizi S, Sullivan EA. *Chlamydia* in the Pacific Region, the Silent Epidemic. Sex Transm Dis. 2008 Sep;35(9):801–806.
- Svigals V, Blair A, Muller S, Sahu Khan A, Faktaufon D, Kama M, et al. Hyperendemic *Chlamydia trachomatis* sexually transmitted infections among females represent a high burden of asymptomatic disease and health disparity among Pacific Islanders in Fiji. PLoS Negl Trop Dis. 2020 Jan 23;14(1):e0008022.
- World Health Organization. Global health sector strategy on sexually transmitted infections, 2016–2021. Available from: https://www.who.int/publications/i/item/WHO-RHR-16.09
- Unemo M, Lahra MM, Escher M, Eremin S, Cole MJ, Galarza P, et al. WHO global antimicrobial resistance surveillance for *Neisseria gonorrhoeae* 2017–18: a retrospective observational study. Lancet Microbe. 2021;2:e627–e636. doi: 10.1016/S2666-5247(21)00171-3
- Vallely LM, Toliman P, Ryan C, Rai G, Wapling J, Gabuzzi J, et al. Performance of syndromic management for the detection and treatment of genital *Chlamydia trachomatis*, *Neisseria gonorrhoeae* and *Trichomonas vaginalis* among women attending antenatal, well woman and sexual health clinics in Papua New Guinea: a cross-sectional study. BMJ Open. 2017 Dec 29;7(12):e018630.
